# Supplementary material for: Niraparib restrains prostate cancer cell proliferation and metastasis and tumor growth in mice by regulating the lncRNA MEG3/miR-181-5p/GATA6 pathway
Source: PeerJ. 2023 Nov 29;11:e16314. doi: 10.7717/peerj.16314 (PMC10693232; doi:10.7717/peerj.16314)
Supplement: Supplemental Information 2 [file peerj-11-16314-s002.pptx]

## Slide 1
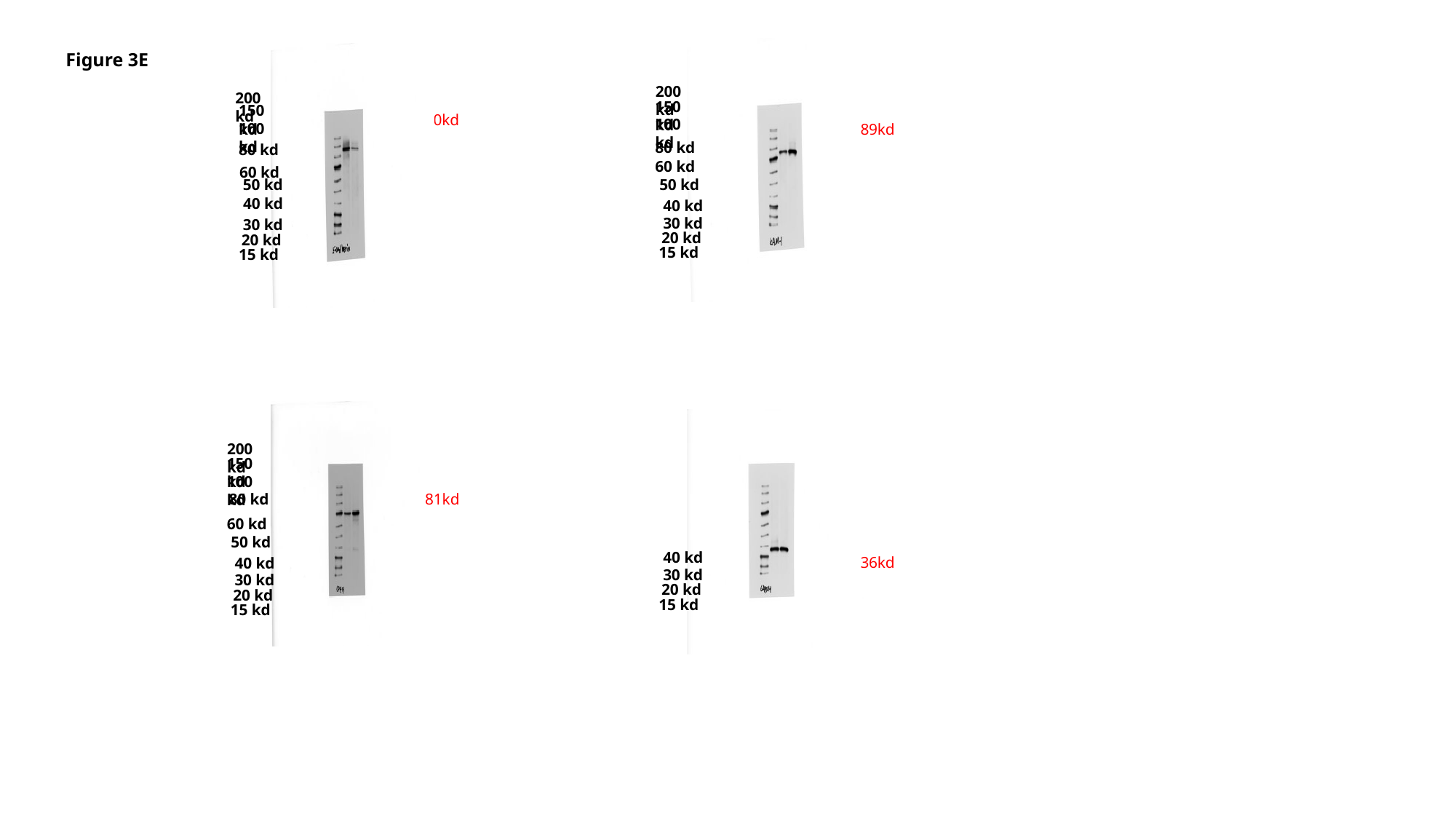

200 kd
150 kd
100 kd
89kd
80 kd
60 kd
50 kd
40 kd
30 kd
20 kd
15 kd
Figure 3E
200 kd
150 kd
100 kd
80 kd
60 kd
50 kd
40 kd
30 kd
20 kd
15 kd
120kd
200 kd
150 kd
100 kd
80 kd
81kd
60 kd
50 kd
40 kd
30 kd
20 kd
15 kd
40 kd
36kd
30 kd
20 kd
15 kd

## Slide 2
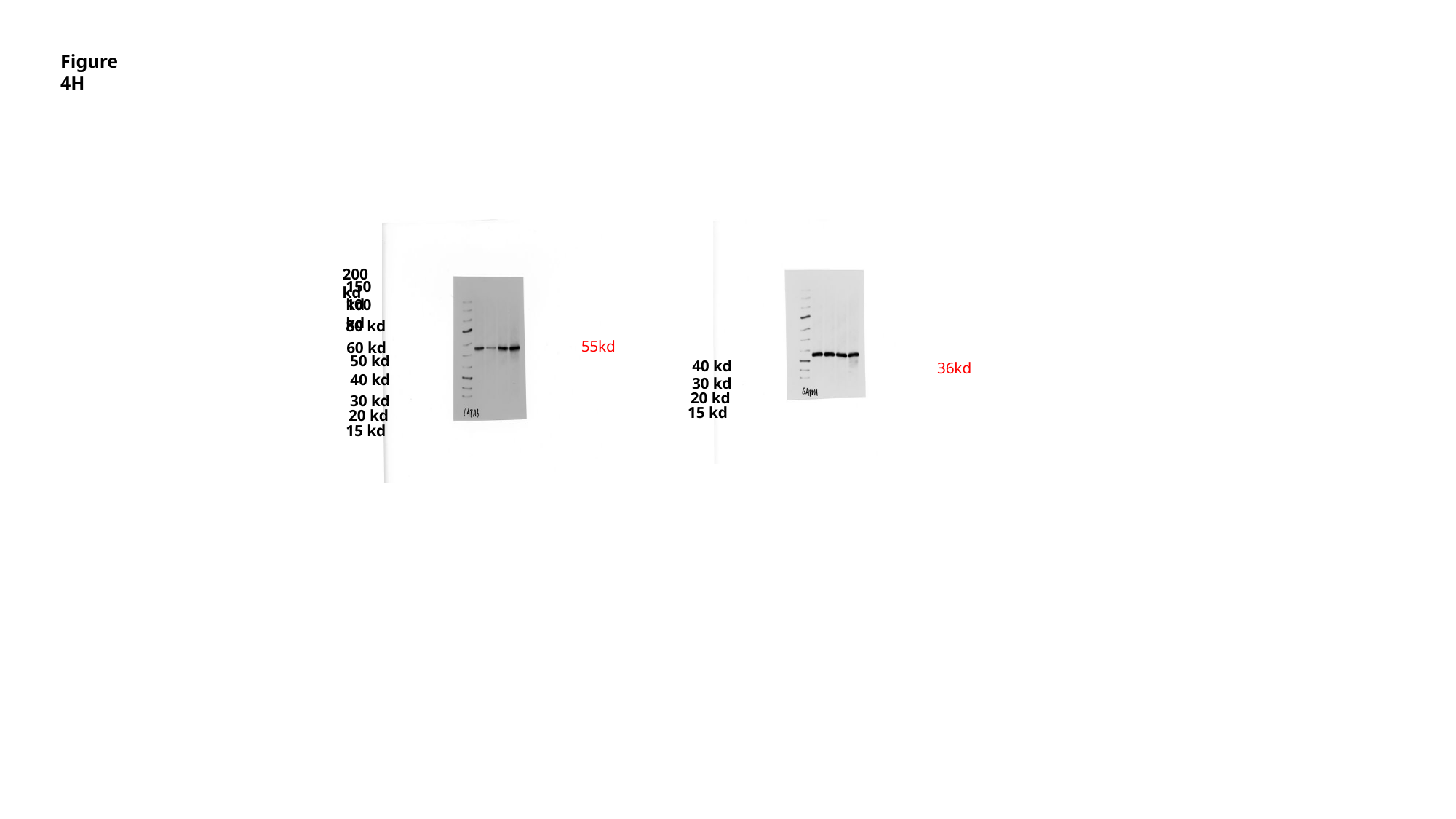

Figure 4H
200 kd
150 kd
100 kd
80 kd
60 kd
50 kd
40 kd
30 kd
20 kd
15 kd
40 kd
36kd
30 kd
20 kd
15 kd
55kd

## Slide 3
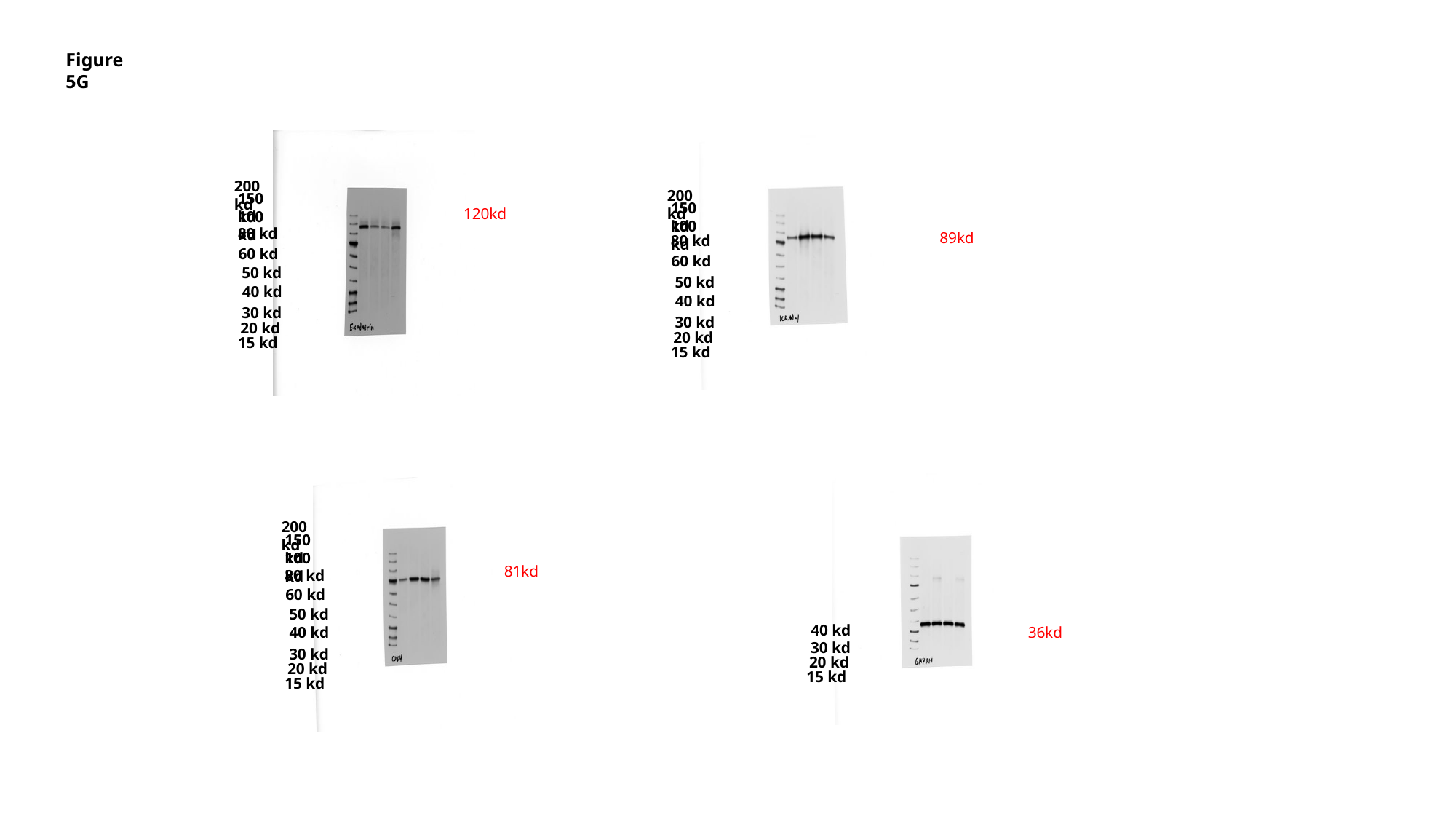

Figure 5G
200 kd
150 kd
120kd
100 kd
80 kd
60 kd
50 kd
40 kd
30 kd
20 kd
15 kd
200 kd
150 kd
100 kd
89kd
80 kd
60 kd
50 kd
40 kd
30 kd
20 kd
15 kd
40 kd
36kd
30 kd
20 kd
15 kd
200 kd
150 kd
100 kd
80 kd
60 kd
50 kd
40 kd
30 kd
20 kd
15 kd
81kd

## Slide 4
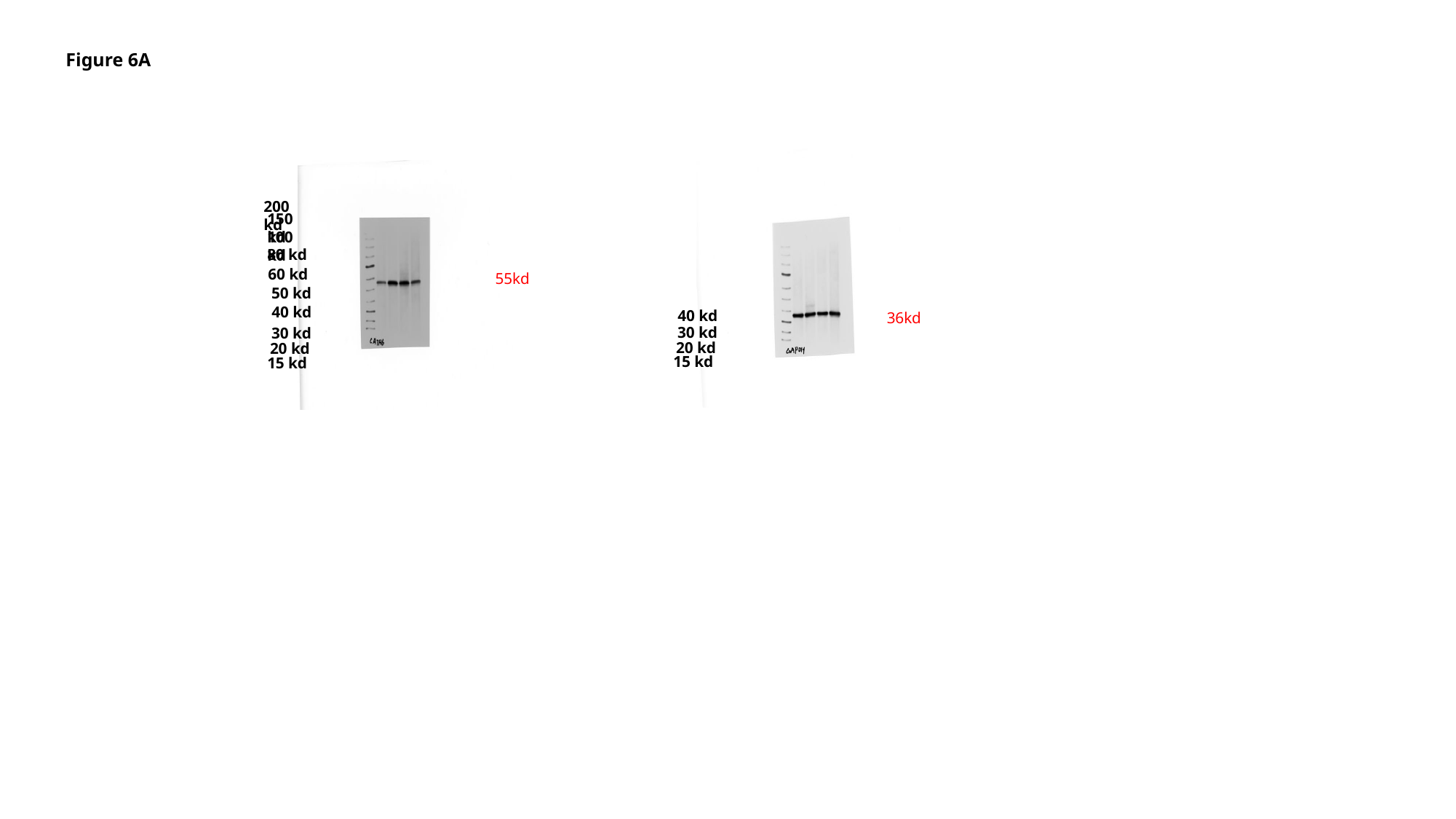

Figure 6A
40 kd
36kd
30 kd
20 kd
15 kd
200 kd
150 kd
100 kd
80 kd
60 kd
55kd
50 kd
40 kd
30 kd
20 kd
15 kd

## Slide 5
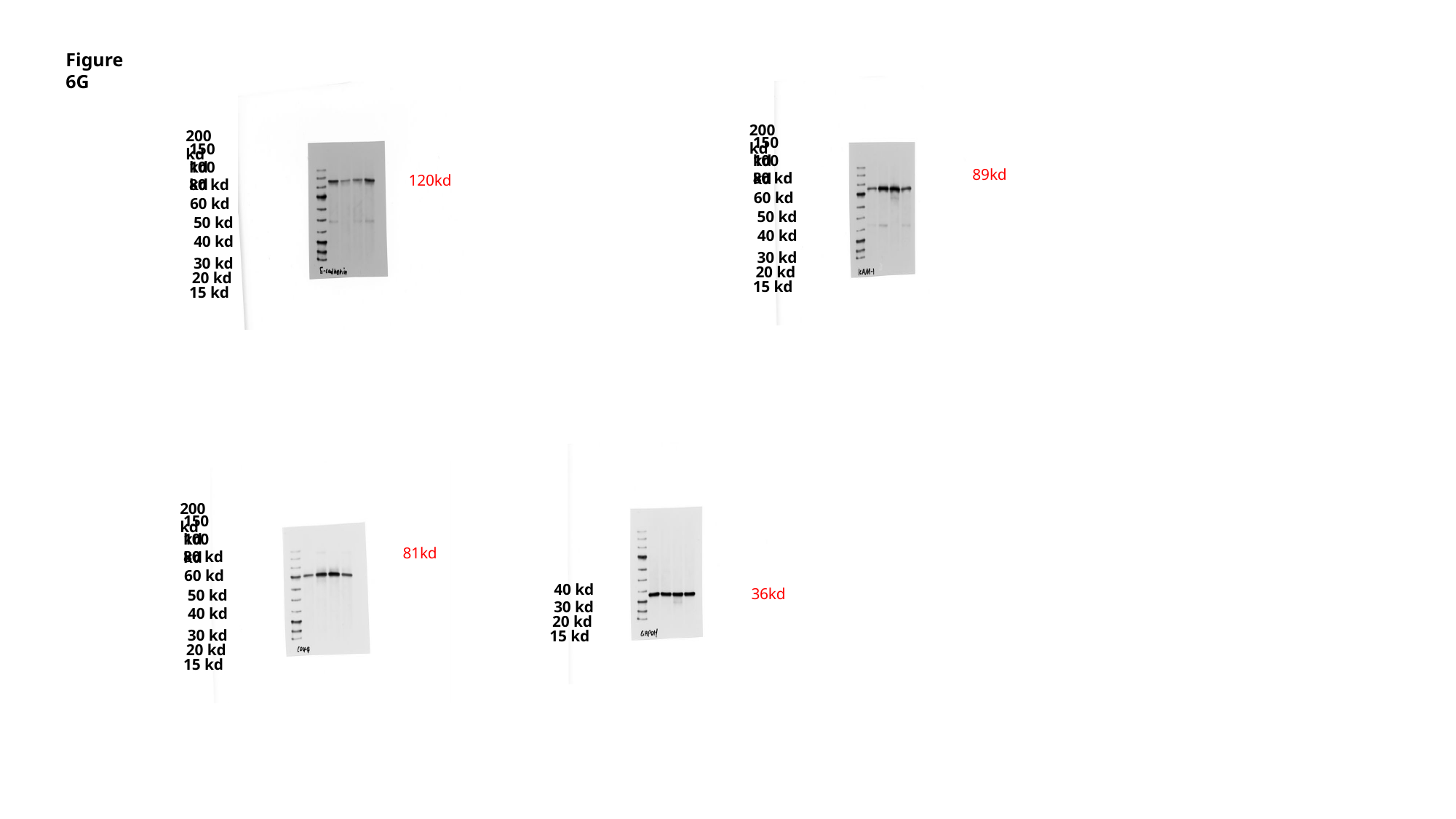

Figure 6G
200 kd
150 kd
100 kd
89kd
80 kd
60 kd
50 kd
40 kd
30 kd
20 kd
15 kd
200 kd
150 kd
100 kd
120kd
80 kd
60 kd
50 kd
40 kd
30 kd
20 kd
15 kd
40 kd
36kd
30 kd
20 kd
15 kd
200 kd
150 kd
100 kd
81kd
80 kd
60 kd
50 kd
40 kd
30 kd
20 kd
15 kd

## Slide 6
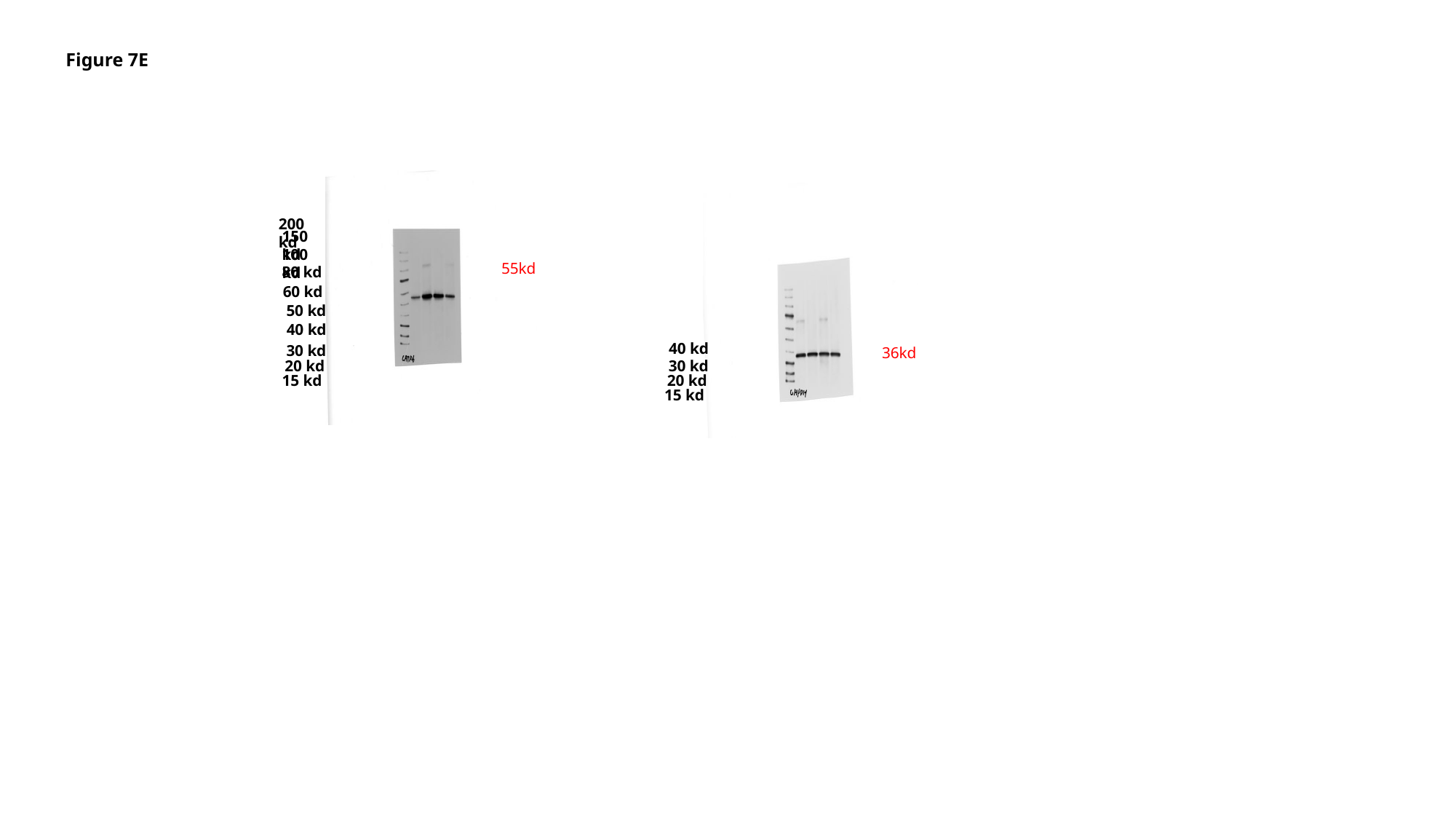

Figure 7E
200 kd
150 kd
100 kd
55kd
80 kd
60 kd
50 kd
40 kd
30 kd
20 kd
15 kd
40 kd
36kd
30 kd
20 kd
15 kd
