# Supplementary material for: Niraparib restrains prostate cancer cell proliferation and metastasis and tumor growth in mice by regulating the lncRNA MEG3/miR-181-5p/GATA6 pathway
Source: PeerJ. 2023 Nov 29;11:e16314. doi: 10.7717/peerj.16314 (PMC10693232; doi:10.7717/peerj.16314)
Supplement: Supplemental Information 4 [file peerj-11-16314-s004.pdf]

Table 1. MIQE checklist for authors, reviewers, and editors.[a]

Item to check	Section	Item to check	Section
Experimental design		qPCR oligonucleotides	
Definition of experimental and control groups	Section 3	Primer sequences	Section 2.4
Number within each group	Figure legends	RTPrimerDB identification number	Not applicable
Assay carried out by the core or investigator's laboratory?	investigator	Probe sequences	Not applicable
Acknowledgment of authors' contributions	Statement	Location and identity of any modifications	Not applicable
Sample		Manufacturer of oligonucleotides	Not applicable
Description	Section 2.4	Purification method	Not applicable
Volume/mass of sample processed	Section 2.4	qPCR protocol	
Microdissection or macrodissection	Not applicable	Complete reaction conditions	Section 2.4
Processing procedure	Section 2.4	Reaction volume and amount of cDNA/DNA	Section 2.4
If frozen, how and how quickly?	Section 2.4	Primer, (probe), Mg^{2+}, and dNTP concentrations	Section 2.4
If fixed, with what and how quickly?	Not applicable	Polymerase identity and concentration	Section 2.4
Sample storage conditions and duration (especially for FFPE[b] samples)		Buffer/kit identity and manufacturer	Section 2.4
Nucleic acid extraction		Exact chemical composition of the buffer	Not applicable
Procedure and/or instrumentation	Section 2.4	Additives (SYBR Green I, DMSO, and so forth)	Section 2.4
Name of kit and details of any modifications	Section 2.4	Manufacturer of plates/tubes and catalog number	Not applicable
Source of additional reagents used	Not applicable	Complete thermocycling parameters	Section 2.4
Details of DNase or RNase treatment	Section 2.4	Reaction setup (manual/robotic)	Not applicable
Contamination assessment (DNA or RNA)	Section 2.4	Manufacturer of qPCR instrument	Section 2.4
Nucleic acid quantification	Section 2.4	qPCR validation	
Instrument and method	Section 2.4	Evidence of optimization (from gradients)	Not applicable
Purity (A_{260}/A_{280})	Not applicable	Specificity (gel, sequence, melt, or digest)	Section 2.4
Yield	Not applicable	For SYBR Green I, C_q of the NTC	Not applicable
RNA integrity: method/instrument	Section 2.4	Calibration curves with slope and *y* intercept	Section 2.4
RIN/RQI or C_q of 3′ and 5′ transcripts	Section 2.4	PCR efficiency calculated from slope	Not applicable
Electrophoresis traces	Not applicable	CIs for PCR efficiency or SE	Not applicable
Inhibition testing (C_q dilutions, spike, or other)	Section 2.4	r^2 of calibration curve	Not applicable
Reverse transcription		Linear dynamic range	Not applicable
Complete reaction conditions	Section 2.4	C_q variation at LOD	Not applicable
Amount of RNA and reaction volume	Section 2.4	CIs throughout range	Not applicable
Priming oligonucleotide (if using GSP) and concentration	Section 2.4	Evidence for LOD	Not applicable
Reverse transcriptase and concentration	Section 2.4	If multiplex, efficiency and LOD of each assay	Not applicable
Temperature and time	Section 2.4	Data analysis	
Manufacturer of reagents and catalogue numbers	Not applicable	qPCR analysis program (source, version)	Section 2.4
C_qs with and without reverse transcription	Not applicable	Method of C_q determination	Not applicable
Storage conditions of cDNA	Not applicable	Outlier identification and disposition	Not applicable
qPCR target information		Results for NTCs	Not applicable
Gene symbol	Not applicable	Justification of number and choice of reference genes	Section 2.4
Sequence accession number	Not applicable	Description of normalization method	Section 2.4
Location of amplicon	Not applicable	Number and concordance of biological replicates	Not applicable
Amplicon length	Not applicable	Number and stage (reverse transcription or qPCR) of technical replicates	Section 2.4
In silico specificity screen (BLAST, and so on)	Not applicable	Repeatability (intraassay variation)	Section 2.12
Pseudogenes, retropseudogenes, or other homologs?	Not applicable	Reproducibility (interassay variation, CV)	Not applicable
Sequence alignment	Not applicable	Power analysis	Not applicable
Secondary structure analysis of amplicon	Not applicable	Statistical methods for results significance	Section 2.12
Location of each primer by exon or intron (if applicable)	Not applicable	Software (source, version)	Section 2.12
What splice variants are targeted?	Not applicable	C_q or raw data submission with RDML	Not applicable

[a] All essential information (E) must be submitted with the manuscript. Desirable information (D) should be submitted if available. If primers are from RTPrimerDB, information on qPCR target, oligonucleotides, protocols, and validation is available from that source.
[b] FFPE, formalin-fixed, paraffin-embedded; RIN, RNA integrity number; RQI, RNA quality indicator; GSP, gene-specific priming; dNTP, deoxynucleoside triphosphate.
[c] Assessing the absence of DNA with a no–reverse transcription assay is essential when first extracting RNA. Once the sample has been validated as DNA free, inclusion of a no–reverse transcription control is desirable but no longer essential.
[d] Disclosure of the probe sequence is highly desirable and strongly encouraged; however, because not all vendors of commercial predesigned assays provide this information, it cannot be an essential requirement. Use of such assays is discouraged.
